# L-shaped association between lean body mass to visceral fat mass ratio with hyperuricemia: a cross-sectional study

**DOI:** 10.1186/s12944-024-02111-2

**Published:** 2024-04-20

**Authors:** Longti Li, Ya Shao, Huiqin Zhong, Yu Wang, Rong Zhang, Boxiong Gong, Xiaoxv Yin

**Affiliations:** 1 Department of Social Medicine and Health Management, School of Public Health, Tongji Medical College, Huazhong University of Science and Technology, No. 13 Hangkong Road, Wuhan, Hubei 430030 PR China; 2 Innovation Centre of Nursing Research, TaiHe Hospital, Hubei University of Medicine, Shiyan, Hubei PR China; 3 Health Management Center, Wudangshan Campus, TaiHe Hospital, Hubei University of Medicine, Shiyan, Hubei PR China

**Keywords:** Lean body mass, Visceral fat mass, Hyperuricemia, NHANES

## Abstract

**Background:**

Insufficient attention has been given to examining the correlation between body composition and hyperuricemia, leading to inconsistent findings. The primary objective of this research is to explore the association between lean body mass index (LMI), visceral fat mass index (VFMI), and hyperuricemia. A specific emphasis will be placed on assessing the link between the ratio of lean body mass to visceral fat mass (LMI/VFMI) and hyperuricemia.

**Methods:**

The present study employed a cross-sectional design and involved a total of 9,646 individuals who participated in the National Health and Nutrition Examination Survey (NHANES). To explore the associations among the variables, logistic and linear regressions were employed. Additionally, subgroup analyses and sensitivity analyses were conducted based on various characteristics.

**Results:**

The results showed that LMI was positively associated with hyperuricemia (for Per-SD: OR = 1.88, 95%CI: 1.75, 2.01; for quartiles [Q4:Q1]: OR = 5.37, 95%CI: 4.31, 6.69). Meanwhile, VFMI showed a positive association with hyperuricemia (for Per-SD: OR = 2.02, 95%CI: 1.88, 2.16; for quartiles [Q4:Q1]: OR =8.37, 95%CI: 6.70, 10.47). When considering the effects of In LMI/VFMI, an L-shaped negative association with hyperuricemia was observed (for Per-SD: OR = 0.45, 95%CI: 0.42, 0.49; for quartiles [Q4:Q1]: OR = 0.16, 95%CI: 0.13, 0.20). Subgroup and sensitivity analyses demonstrated the robustness of this association across different subgroups. Additionally, the segmented regression analysis indicated a saturation effect of 5.64 for the In LMI/VFMI with hyperuricemia (OR = 0.20, 95%CI: 0.17, 0.24). For every 2.72-fold increase of In LMI/VFMI, the risk of hyperuricemia was reduced by 80%.

**Conclusion:**

The LMI/VFMI ratio is non-linearly associated with serum uric acid. Whether this association is causal needs to be confirmed in further longitudinal studies or Mendelian randomization.

**Supplementary Information:**

The online version contains supplementary material available at 10.1186/s12944-024-02111-2.

## Introduction

Globally, hyperuricemia is on the rise, posing a significant health threat, as evidenced by a recent U.S. study reported that 20% of adults aged 20 or older had hyperuricemia [1]. Similarly, another survey conducted in China among adults aged 18 to 59 reported a prevalence rate of hyperuricemia at 15% [2]. Health outcomes across a wide variety of diseases are robustly correlated with hyperuricemia [3], encompassing but not restricted to hypertension [4], diabetes mellitus [5], cardiovascular and cerebrovascular disease [6], and all-cause mortality [7]. Consequently, it is imperative to ascertain the factors linked to hyperuricemia.

Among the risk factors for hyperuricemia, obesity is an important one. Researchers have investigated the connection between hyperuricemia and conventional body metrics like waist circumference (WC) and body mass index (BMI) [8, 9]. Moreover, researchers have examined the correlation between other alternative indicators for assessing obesity and hyperuricemia, such as lipid accumulation product, body roundness index, and visceral adiposity index [10–12]. However, these proxies are derived indirectly from physical measurements or a combination of physical measures (such as BMI, WC, or height) and blood markers (such as triglycerides or high-density lipoprotein cholesterol). Accordingly, they do not facilitate a comprehensive and precise visual assessment of obesity severity and body fat distribution across the entire body.

Recent progress has been made in assay methodologies for assessing body composition [13]. These new methods offer enhanced precision in discerning muscle and adipose tissue distribution. Many investigations have substantiated the adverse influence of adipose tissue on hyperuricemia [14, 15]. However, it must be acknowledged that there may also be some degree of association between muscle tissue and serum uric acid (SUA) levels, a relationship confirmed by Chen et al. [16]. Exploring the correlation between adipose tissue and hyperuricemia in isolation may be confounded by other tissues in the body composition, especially muscle tissue. This construct has usually yet to be considered in previous research.

Contemporary investigations have further revealed that maintaining an optimal proportion of lean body mass to adipose tissue yields advantageous outcomes in mitigating metabolic risk [17]. We are dedicated to researching the relationship between body composition and metabolic health [18, 19]. However, the existing evidence is inadequate to establish a correlation between the proportion of lean body mass to visceral fat and hyperuricemia. Consequently, this specific association was the primary objective of our research.

## Material and methods

### Study design

This cross-sectional study comprised four cycles of the National Health and Nutrition Examination Survey (NHANES) conducted in the U.S. between 2011 and 2018. Approximately 5,000 individuals were selected from 15 countries across the U.S. each year to participate in the survey. For more information about the survey, see the NHANES Plan and Operations manual [20]. The National Center for Health Statistics Research Ethics Review Board approved the survey (Protocol #2011-17 and Protocol #2018-01). The present study is not subject to ethical review as a secondary analysis of information from that survey.

### Study population

**Fig. 1:**
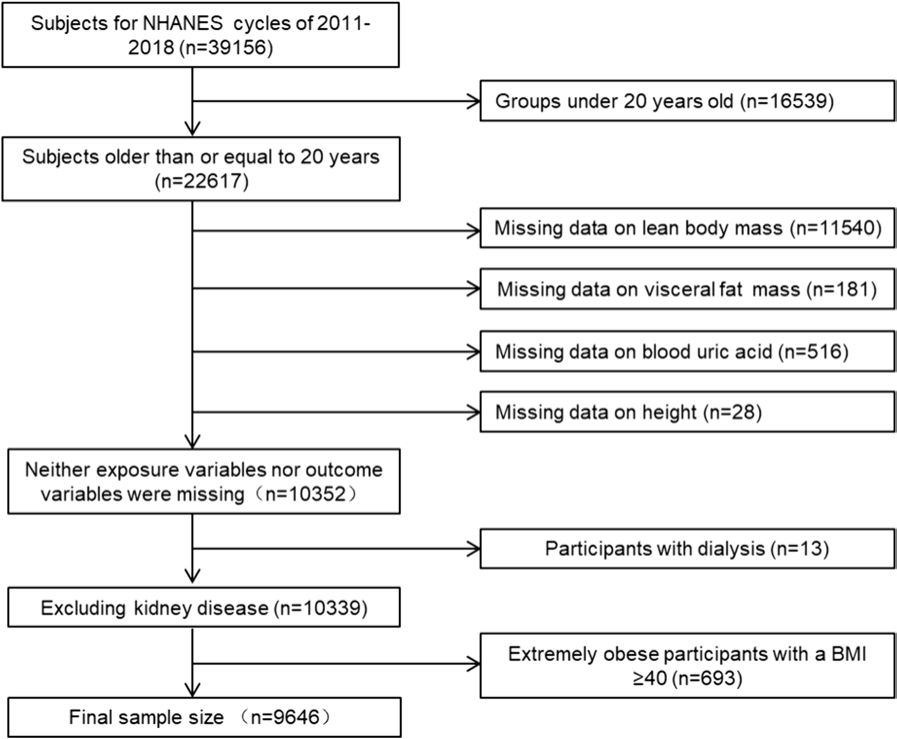
Flowchart for the selection of subjects

In the period spanning from 2011 to 2018, 22,617 individuals over 20 years participated in four cycles. Among these participants, 10,896 completed the body composition assessment, while 10,380 completed the SUA measurement. Twenty-eight individuals were excluded because they were missing height information to calculate standardized indexes of lean body and visceral fat mass. To account for potential confounding factors of renal disease, we excluded individuals who underwent dialysis the previous year and had an estimated glomerular filtration rate (eGFR) lower than 30 ml/min/1.73m2. Additionally, individuals who are obese with a BMI exceeding 40 kg/m^2^ were also excluded, as this population is known to experience a multitude of metabolic disorders that may interfere with the study outcomes. According to the abovementioned criteria, a cumulative count of 9,646 study participants remained for further evaluation. The graphical representation of participant inclusion can be observed in Fig. 1. This study is reported in accordance with the STROBE statement (Supplementary File S1).

### Measurement of lean and visceral fat mass

Lean and visceral fat mass were measured using Dual-Energy X-ray Absorptiometry in a mobile examination center, where lean body mass excluded bone mineral content. In the NHANES survey, individuals aged 8-59 were eligible for the screening, with exclusions for pregnancy, recent ingestion of radiographic material, and individuals weighing over 450 pounds or were over 6 feet 5 inches tall. Before the test, participants were instructed to remove all metal objects from their bodies. The scanning process follows stringent quality control procedures, beginning with the involvement of trained and certified radiologic technologists who scan all exams. The Hologic Anthropomorphic Spine Phantom in the mobile examination canter was scanned daily to ensure accurate equipment calibration. Additionally, the NHANES Quality Control Center conducts expert reviews of all participant scans to ensure consistency of results [21]. Considering the potential effect of height on the variables, we calculated the lean body mass index (LMI) and visceral fat mass index (VFMI) following the common practice of previous related studies [22].

### Hyperuricemia assessment

SUA was measured after participants collected blood samples at the mobile examination center, and the samples were cryogenically stored until transported to the collaborating laboratory for analysis. Standardized trained technicians tested SUA concentrations using the Beckman Coulter UniCel® DxC800 (2011-2016) and the Roche Cobas 6000 (2017-2018) [23]. Hyperuricemia was initially identified as SUA levels exceeding 7.0 mg/dL in men and 6.0 mg/dL in women [24]. However, due to the ongoing debate regarding the appropriate cutoff value for elevated SUA levels, we performed a sensitivity analysis employing a cutoff value of SUA ≥6.8 mg/dL. This particular value was chosen as it aligns with the solubility of uric acid under normal physiological pH and temperature conditions [25].

### Covariates

Based on prior knowledge and existing literature [26, 27], a broad range of confounders were considered, including sex, age, race, education level, poverty-to-income ratio (PIR), WC, and BMI. A health technician measured WC and BMI at a mobile examination center. Participants were divided into three groups based on BMI: normal weight (BMI<25), overweight (25≤BMI<30), and obese (BMI≥30). WC was classified as healthy (male WC <94, female WC <80) and unhealthy (male WC ≥94, female WC ≥80) [28]. Additionally, activity, smoking, and alcohol were also taken into account. We assessed participants' activity levels using metabolic equivalent (MET) scores, calculated by quantifying the time they spent each week engaged in a range of work-related vigorous/moderate activities, amateur physical activity, and walking or cycling. Individuals with MET scores < 600 per week were defined as having low activity levels; scores between 600-3000 indicated moderate, and higher than 3000 were identified as vigorous [29]. The evaluation of smoking and alcohol consumption was conducted through Alcohol and Cigarette Use Questionnaires. A 'Current smoker' was identified as someone who has smoked 100 or more cigarettes in the past and does so now [30]. Alcohol was categorized based on consuming more than 12 drinks per year [31].

The present study evaluated the main disease statuses, including hypertension, diabetes, cardiovascular disease (CVD), and gout. Hypertension was operationally defined as being previously diagnosed by a healthcare professional, involving antihypertensive medication, or exhibiting systolic or diastolic blood pressure that exceeds the current recommended standard (140/90 mmHg) [32]. Diabetes mellitus was defined by a prior diagnosis, glucose-lowering medications or insulin treatment, fasting blood glucose, 2-hour oral glucose tolerance tests, or glycated hemoglobin above the current diagnostic criteria [33]. CVD was determined based on a medical status questionnaire, including whether a doctor or health-related professional had told participants to have heart failure, coronary heart disease, angina/angina pectoris, heart attack, or stroke. Gout was achieved through a questionnaire that asked participants whether they had been previously diagnosed by healthcare workers. In addition, blood markers, including total cholesterol (TC), alanine aminotransferase (ALT), and eGFR were also considered. The eGFR was used in the CKD-EPI 2021 creatinine equation [34].

A directed acyclic graph (DAG) was utilized to pinpoint potential covariates that required adjustment in the multivariable analysis [35]. Referring to the DAG (see supplementary figure 1), a minimal set of variables was selected for adjustment: sex, age, race, education level, PIR, physical activity, alcohol consumption, and smoking.

### Statistical analysis

The mean ± standard deviation (SD) represents the characteristics of the participating population that adhere to the normal distribution. At the same time, the median and interquartile range was used to describe those characteristics that deviate from normality. Percentages were used to report categorical variables. Multivariable logistic regression models were applied to investigate the associations between LMI, VFMI, and the ratio of LMI to VFMI with hyperuricemia. To account for the skewed distribution of LMI/VFMI, we applied a natural logarithmic transformation (In LMI/VFMI) to ensure its normal distribution. Before the regression analysis, a diagnostic assessment of multicollinearity was conducted to identify any issues about covariance among the independent variables. A variance inflation factor value below 10 indicates acceptable levels of multicollinearity [36]. The missing covariate data were estimated using the chained equation method of multiple imputations (MICE), and a total of five imputed datasets were created.

Three models were used in the regression analysis. Model 1 did not adjust for any variables, model 2 adjusted for gender, age, and race, and model 3 adjusted for sex, age, race, education level, PIR, MET scores, alcohol, and smoking. An evaluation of the dose-response relationship between exposure variables and hyperuricemia was carried out using generalized additive models (with a logit link), a method widely used to evaluate non-linear relationships between variables [37, 38]. Threshold effects between In LMI/VFMI and hyperuricemia were analyzed using smoothed curve fitting. The specific methods were segmented regression, which involved utilizing separate lines to fit each interval [39]. The segmented regression model was compared with the single-line model through log-likelihood ratio tests to identify the presence of a critical point.

The stability of the results was verified with subgroup analyses and sensitivity analyses. Subgroup analyses were conducted with separate stratification for different genders, BMI, WC, MET score, smoking, alcohol consumption, hypertension, diabetes mellitus, CVD, gout, and eGFR. We examined five primary scenarios in our sensitivity analyses: varying hyperuricemia thresholds, evaluating SUA as a continuous variable, analyzing the raw data without implementing MICE, variations in methods of SUA testing across different cycles, and considering the potential use of SUA lowering drugs by patients with gout.

## Results

### Characteristics of participants

Out of the 9,646 study subjects, 1,455 were diagnosed with hyperuricemia. Subjects averaged 39 years of age, and 49.4% were female. A statistical analysis revealed significant differences in various aspects of the groups according to the quartiles of In LMI/VFMI, including gender, race, education level, MET scores, smoking, alcohol, gout, hypertension, diabetes, CVD, and SUA. In the highest quartile of In LMI/VFMI, age, BMI, WC, TC, and ALT were lower. More detailed information can be found in Table 1.

**Table 1:** Baseline characteristics of participants according to the quartiles of lean body mass to visceral fat mass ratio (*n* = 9646)

**Characteristic**	**In LMI/VFMI**				***P*-value**
	Q1 (<4.41)	Q2 (4.41-4.76)	Q3 (4.76-5.17)	Q4 (≥5.17)	
**Gender, n (%)**					<0.001
Male	816 (33.84)	1298 (53.81)	1373 (56.95)	1395 (57.84)	
Female	1595 (66.16)	1114 (46.19)	1038 (43.05)	1017 (42.16)	
**Age (mean ± SD, year)**	46.54 ± 9.41	41.54 ± 10.27	37.22 ± 10.82	31.63 ± 9.87	<0.001
**Race, n (%)**					<0.001
Mexican American	566 (23.48)	426 (17.66)	287 (11.90)	142 (5.89)	
Non-Hispanic White	844 (35.01)	819 (33.96)	791 (32.81)	900 (37.31)	
Non-Hispanic Black	228 (9.46)	398 (16.50)	559 (23.19)	745 (30.89)	
Other Race	773 (32.06)	769 (31.88)	774 (32.10)	625 (25.91)	
**Education, n (%)**^a^					<0.001
Under high school	601 (24.93)	496 (20.56)	387 (16.06)	288 (11.95)	
High school or equivalent	539 (22.36)	534 (22.14)	528 (21.91)	481 (19.95)	
Above high school	1271 (52.72)	1382 (57.30)	1495 (62.03)	1642 (68.10)	
**PIR (mean ± SD)**^a^	2.43 ± 1.65	2.60 ± 1.65	2.63 ± 1.67	2.54 ± 1.67	<0.001
**BMI (mean ± SD, kg/m**^**2**^**)**	30.70 ± 4.49	28.93 ± 4.54	27.10 ± 4.70	23.94 ± 4.43	<0.001
**WC (mean ± SD, cm) **^a^	103.38 ± 11.30	98.75 ± 11.24	93.36 ± 11.80	83.29 ± 11.00	<0.001
**MET scores, n (%)**^a^					<0.001
Light	387 (22.49)	324 (16.62)	263 (13.01)	198 (9.25)	
Moderate	691 (40.15)	782 (40.12)	797 (39.42)	784 (36.64)	
Vigorous	643 (37.36)	843 (43.25)	962 (47.58)	1158 (54.11)	
**Smoking, n (%)**^a^					<0.001
Never	740 (59.97)	773 (60.49)	760 (61.69)	791 (64.62)	
Former	248 (20.10)	221 (17.29)	193 (15.67)	148 (12.09)	
Current	246 (19.94)	284 (22.22)	279 (22.65)	285 (23.28)	
**Alcohol, n (%)**^a^					0.498
Yes	1472 (97.35)	1676 (97.44)	1718 (97.72)	1828 (98.07)	
No	40 (2.65)	44 (2.56)	40 (2.28)	36 (1.93)	
**TC (median (IQR), mg/dL)**	201.00 (175.00-228.00)	193.50 (169.00-220.00)	186.00 (163.00-212.00)	171.00 (152.00-194.25)	<0.001
**ALT (median (IQR), U/L)**	23.00 (17.00-34.00)	22.00 (16.00-31.00)	20.00 (15.00-29.00)	18.00 (14.00-24.00)	<0.001
**eGFR (median (IQR), ml/min/1.73m**^**2**^**)**	104.95 (92.82-113.18)	105.38 (92.43-115.81)	106.41 (92.88-117.39)	107.12 (93.39-119.31)	<0.001
**Gout, n (%)**^a^					<0.001
Yes	90 (3.73)	66 (2.74)	39 (1.62)	21 (0.87)	
No	2321 (96.27)	2344 (97.26)	2370 (98.38)	2391 (99.13)	
**Hypertension, n (%)**					<0.001
Yes	965 (40.02)	680 (28.19)	498 (20.66)	277 (11.48)	
No	1446 (59.98)	1732 (71.81)	1913 (79.34)	2135 (88.52)	
**Diabetes, n (%)**					<0.001
Yes	509 (21.11)	254 (10.53)	126 (5.23)	49 (2.03)	
No	1902 (78.89)	2158 (89.47)	2285 (94.77)	2363 (97.97)	
**CVD, n (%)**					<0.001
Yes	159 (6.59)	84 (3.48)	58 (2.41)	38 (1.58)	
No	2252 (93.41)	2328 (96.52)	2353 (97.59)	2374 (98.42)	
**SUA (mean ± SD, mg/dL)**	5.35 ± 1.35	5.43 ± 1.43	5.33 ± 1.39	5.00 ± 1.26	<0.001
**LMI (median (IQR), kg/m**^**2**^**)**	18.07 (16.15-19.89)	18.32 (16.17-20.29)	17.75 (15.54-19.90)	16.71 (14.73-18.98)	<0.001
**VFMI (median (IQR), kg/m**^**2**^**)**	0.27 (0.24-0.32)	0.18 (0.16-0.21)	0.13 (0.11-0.15)	0.07 (0.06-0.09)	<0.001
**In LMI/VFMI**	4.21 (4.05-4.32)	4.59 (4.50-4.68)	4.95 (4.85-5.05)	5.46 (5.31-5.65)	<0.001

*Abbreviations*: *LMI* Lean body mass index, *VFMI* Visceral fat mass index, *SD* Standard deviation, *IQR* Interquartile range, *PIR* Poverty income ratio, *BMI* Body mass index, *WC* Waist circumference, *TC* Total cholesterol, *ALT* Alanine aminotransferase, *eGFR* Estimated glomerular filtration rate, *CVD* Cardiovascular disease, *SUA* Serum uric acid

^a^The following variables had missing information: education level (0.02%), PIR (8.07%), WC (0.57%), MET scores (18.81%), smoking (48.50%), alcohol (28.94%), gout (0.04%)

### Association of In LMI/VFMI with hyperuricemia

Table 2 illustrates the logistic regression results. The regression analyses, whether unadjusted, partially adjusted, or fully adjusted, consistently demonstrated a positive association between LMI and hyperuricemia. The OR for Per- SD was 1.88 with a 95% CI of 1.75 to 2.01, while the OR for quartile 4 versus quartile 1 was 5.37 with a 95% CI of 4.31 to 6.69.

**Table 2:** Multiple logistics regression analysis for the relationship between lean body mass index, visceral fat mass index and lean body mass to visceral fat mass ratio with hyperuricemia

**Independent variables**	**Model 1 (*n*=9646)**		**Model 2 (*n*=9646)**		**Model 3 (*n*=9646)**	
	OR (95%CI)	*P*-value	OR(95%CI)	*P*-value	OR (95%CI)	*P*-value
**LMI**						
Per-SD	1.91 (1.80, 2.02)	<0.001	1.96 (1.85, 2.09)	<0.001	1.88 (1.75, 2.01)	<0.001
Q1	1.00 (ref)		1.00 (ref)		1.00 (ref)	
Q2	1.90 (1.54, 2.35)	<0.001	1.98 (1.60, 2.45)	<0.001	1.81 (1.46, 2.26)	<0.001
Q3	3.07 (2.51, 3.75)	<0.001	3.19 (2.61, 3.91)	<0.001	2.80 (2.25, 3.47)	<0.001
Q4	5.91 (4.88, 7.15)	<0.001	6.44 (5.30, 7.82)	<0.001	5.37 (4.31, 6.69)	<0.001
*P* for trend	<0.001		<0.001		<0.001	
**VFMI**						
Per-SD	1.59 (1.51, 1.67)	<0.001	1.87 (1.75, 1.99)	<0.001	2.02 (1.88, 2.16)	<0.001
Q1	1.00 (ref)		1.00 (ref)		1.00 (ref)	
Q2	2.18 (1.78, 2.68)	<0.001	2.52 (2.05, 3.10)	<0.001	2.54 (2.06, 3.13)	<0.001
Q3	3.32 (2.73, 4.03)	<0.001	4.44 (3.61, 5.46)	<0.001	4.61 (3.73, 5.69)	<0.001
Q4	4.61 (3.81, 5.58)	<0.001	7.29 (5.87, 9.04)	<0.001	8.37 (6.70, 10.47)	<0.001
*P* for trend	<0.001		<0.001		<0.001	
**Ln LMI/VFMI**						
Per-SD	0.68 (0.64, 0.72)	<0.001	0.58 (0.54, 0.62)	<0.001	0.45 (0.42, 0.49)	<0.001
Q1	1.00 (ref)		1.00 (ref)		1.00 (ref)	
Q2	0.87 (0.75, 1.01)	0.064	0.79 (0.68, 0.91)	<0.001	0.60 (0.51, 0.70)	<0.001
Q3	0.70 (0.61, 0.82)	<0.001	0.58 (0.49, 0.68)	<0.001	0.40 (0.34, 0.48)	<0.001
Q4	0.35 (0.30, 0.42)	<0.001	0.26 (0.21, 0.32)	<0.001	0.16 (0.13, 0.20)	<0.001
*P* for trend	<0.001		<0.001		<0.001	

*Abbreviations*: *OR* Odds Ratio, *SD* Standard deviation, *CI* Confidence interval, *LMI* Lean body mass index, *VFMI* Visceral fat mass index

Model 1: no variables adjusted. Model 2: sex, age and race were adjusted. Model 3: sex, age, race, education level, PIR, MET scores, alcohol consumption, and smoking were adjusted

At the same time, a positive association was observed between VFMI and hyperuricemia. This association remained significant when VFMI was examined as a continuous and categorical variable. The ORs for Per-SD was 2.02 (95% CI: 1.88, 2.16), and those for quartile 2, quartile 3, and quartile 4 were 2.54 (95% CI: 2.06, 3.13), 4.61 (95% CI: 3.73, 5.69), and 8.37 (95% CI: 6.70, 10.47), respectively, compared to the reference quartile. Furthermore, a significant trend (*P* < 0.001) was observed, indicating an increased risk of hyperuricemia with higher quartiles of VFMI.

A negative correlation was observed when examining the correlation between In LMI/VFMI and hyperuricemia. This correlation remained consistent in the partially or fully adjusted models. We found that with each SD increase in In LMI/VFMI, the risk of hyperuricemia in participants decreased by 55% (OR=0.45; 95% CI: 0.42, 0.49). Likewise, in examining In LMI/VFMI as a categorical variable, a downward trajectory in the prevalence of hyperuricemia was observed as quartiles increased (*P*-value for trend<0.001). The ORs for second, third, and fourth quartile were 0.60 (95% CI: 0.51, 0.70), 0.40 (95% CI: 0.34, 0.48), and 0.16 (95% CI: 0.13, 0.20), respectively.

### Smooth curve fitting and saturation effect analysis

**Fig. 2:**
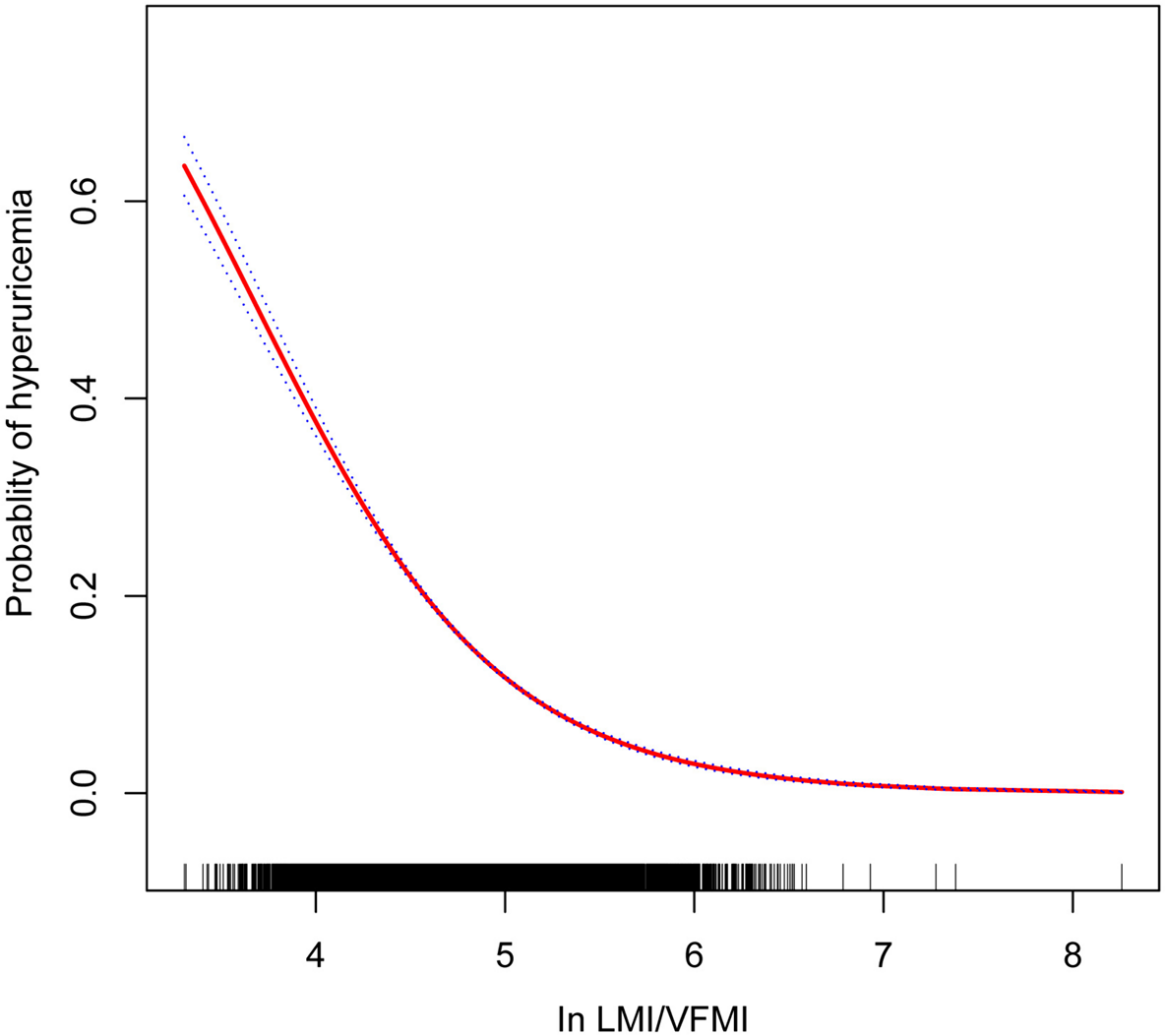
Dose-response relationships between In LMI/VFMI and hyperuricemia. The solid red line depicts a smooth curve. The 95% confidence interval is visualized by the blue bands encompassing the fit

Applying generalized additive modeling in the analysis revealed a non-linear positive association between LMI, VFMI, and hyperuricemia (Supplement Figure 2-3). Upon investigating the association between In LMI/VFMI and hyperuricemia, an L-shaped negative correlation and a saturation effect of 5.64 were identified (Fig. 2). It is worth noting that when In LMI/VFMI was below 5.64, for every 2.72-fold increase in the ratio of LMI to VFMI, the risk of hyperuricemia was reduced by 80% (OR=0.20; 95% CI: 0.17, 0.24). However, once surpassing the critical threshold of 5.64, the connection appeared to stabilize, and the correlation was not statistically meaningful (OR=2.19; 95% CI: 0.86, 5.55). See Table 3.

**Table 3:** Threshold effect analysis of lean body mass to visceral fat mass ratio with hyperuricemia using piece-wise linear regression (*n*=9646)

**Inflection point of Ln LMI/VFMI**	**Effect size (OR)**	**95%CI**	***P*-value**
Model I			
One line effect	0.22	0.19,0.26	<0.001
Model II			
In LMI/VFMI < 5.64	0.20	0.17,0.24	<0.001
In LMI/VFMI ≥ 5.64	2.19	0.86,5.55	0.100
Log-likelihood ratio test			0.001

*Abbreviations*: *OR* Odds ratio, *CI* Confidence interval, *LMI* Lean body mass index, *VFMI* Visceral fat mass index

Adjusted for sex, age, race, education level, PIR, MET scores, alcohol consumption, and smoking

### Subgroup and sensitivity analysis

**Fig. 3:**
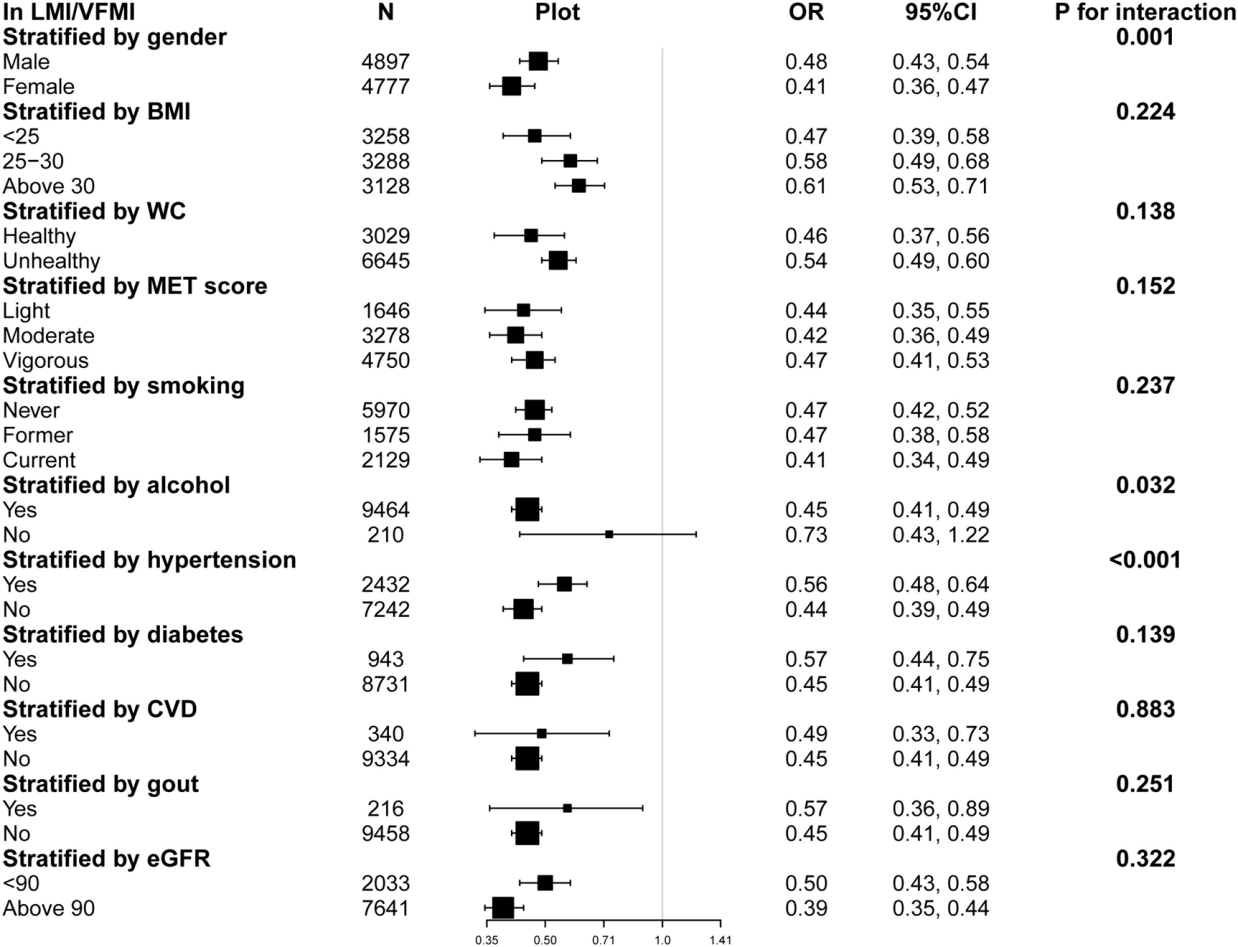
Forest plot of the ORs of hyperuricemia associated with In LMI/VFMI according different subgroups

We performed subgroup analyses to independently evaluate the consistency of the association between the variables related to exposure and hyperuricemia. After adjustment for covariates, the results showed that both LMI and VFMI were significantly and positively associated with hyperuricemia across subgroups (Supplementary Figure 4-5). Across all subgroups, a persistent and steady inverse relationship between In LM/VFM and hyperuricemia was noticed (Fig. 3).

The potential impact of different thresholds for SUA levels on the outcomes was assessed in the sensitivity analyses. Hyperuricemia was defined as SUA levels equal to or greater than 6.8 mmol/L. Furthermore, SUA was also analyzed as a continuous variable. Considering missing data, the original data without MICE was utilized for the analysis. Given the changes in SUA measurements between the years 2017-2018 and the previous cycle, we excluded data from that year in our analyses. Additionally, we excluded gout patients from the data analysis, considering that their SUA levels might have been influenced by medication. The results of these additional analyses did not significantly differ from the previous analyses (Supplement Tables 1-5).

## Discussion

In the current research, a positive association between LMI and VFMI with hyperuricemia was found. Conversely, a negative link was observed when considering the relationship between In LMI/VFMI and hyperuricemia. Regression analyses were conducted, carefully accounting for potential confounders, which allowed us to control for potential biases as much as possible. Additionally, stratified subgroup and sensitivity analyses were performed. The findings revealed that variations in these clinical characteristics did not substantially impact the relationship between the variables, thus affirming the robust and reliable nature of the results.

Scholars have studied the relationship between muscle mass or strength and SUA. However, the available evidence to date could be more varied and inconsistent. A number of researchers have observed a positive correlation between muscle mass or strength and SUA in people of all ages. Alvim et al. [40] discovered that children and adolescents with higher muscle mass also had higher SUA levels. Dong et al. found that elevated SUA was linked to higher muscle mass in community adults over 40 [41]. Similarly, Xu et al. [42] surveyed 992 hospitalized patients over 45 years old and found an inverted J-curve relationship between SUA levels and handgrip strength. According to Nahas et al. [43] and Molino-Lova et al. [44], SUA was positively correlated with muscle strength in older adults. As in the previous study, we similarly found that lean body mass was positively associated with hyperuricemia. However, several other studies have reported contradictory findings. Beavers et al. discovered a strong correlation between high SUA and sarcopenia in a study involving 7,544 adults over 40 from the NHANES III [45]. Similarly, according to a survey of Brazilian adults over 20, muscle mass index was negatively associated with high SUA [46]. Tanaka et al. [47] also found a negative correlation between SUA and skeletal muscle mass in individuals with type 2 diabetes.

There have been several studies examining the link between obesity and hyperuricemia. In China, Han et al. [5] conducted two prospective studies comprising 17,044 individuals who were followed for an average duration of 6 years. Their findings indicated a positive association between BMI and increased SUA levels. A dose-dependent correlation between hyperuricemia and overweight/obesity was illustrated by Choi et al. [48], indicating a population-attributable risk of 44%. Additionally, researchers have examined the link between adipose tissue and SUA by evaluating body composition. A previous investigation reported that the distribution of body fat could potentially impact the occurrence of hyperuricemia among individuals with obesity [49]. Furthermore, Takahashi et al. discovered that visceral adiposity was crucial in increasing SUA concentrations and reducing uric acid clearance. Visceral fat accumulation had a more detrimental impact on uric acid metabolism than BMI and subcutaneous fat accumulation [50]. However, these studies were small sample sizes overall.

Moreover, researchers have discovered that variations in the metabolic outcomes of diverse body fat deposits may exist. In their study, Bai et al. [27] examined a cohort comprising 3,683 individuals who were middle-aged and older. Their findings highlighted a significant association between SUA levels and the presence of visceral and hepatic adipose tissues. However, this correlation was not adequate for subcutaneous fat. Similar findings were observed in other studies [51–53]. In their research, Xie and colleagues [54] examined a sample of 271 children and adolescents in China who were classified as obese. The findings of their study revealed that skeletal muscle emerged as the most significant indicator for hyperuricemia, surpassing both WC and hip circumference. Despite this, no connection was found between hyperuricemia and body fat mass. In a study examining individuals with polycystic ovary syndrome, Zhang and colleagues [55] discovered an unfavorable relationship between SUA and the quantity of visceral adipose tissue. Nevertheless, no substantial association was identified between hyperuricemia and other adipose tissue forms, including overall fat, trunk fat, and subcutaneous abdominal fat. These studies provide valuable insights into the varying effects of different adipose tissues on metabolism, particularly highlighting the significance of visceral fat. This notion is further supported by Li et al.'s study [15]. Their study revealed a significant and positive relationship between SUA and visceral fat area, even among individuals who are not obese (BMI <30 kg/m^2^).

Diminished lean body mass and elevated visceral fat both are strongly linked to an elevated risk of metabolic diseases. When the two are present together, there is likely a synergistic effect on metabolic health [18, 19, 56]. Based on the Chinese National Health Survey, He et al. [57] found that total body fat to muscle ratio was positively correlated with hyperuricemia and that the higher the ratio, the higher the SUA. According to a study conducted by Wang et al. [58], they discovered that the prevalence of hyperuricemia in women, when adjusted for BMI, was positively linked to the ratio of visceral fat area to leg muscle mass. However, this association was not observed in men. Additionally, Zhang et al. [59] examined 5158 Chinese medical check-up records and found a positive relationship between the ratio of visceral fat area to skeletal muscle mass and cardiometabolic diseases. Our current research identified a negative correlation between In LMI/VFMI and hyperuricemia. This negative association was observed across various subgroups, which included stratified analyses based on different sexes, BMI, WC, activity intensity, and disease states.

Changes in body composition occur gradually with age. Muscle mass and strength reach their maximum levels during early adulthood and tend to decline after middle age [60], while muscle mass decreases at 0.75% per year [61]. On the other hand, body fat tends to increase, resulting in visceral fat accumulation and ectopic fat deposition. A longitudinal study conducted by Koster et al. [62] involving 2,307 adults aged 70 and above discovered that increased fat mass was linked to decreased muscle mass. Additionally, surplus fat contributed to a rapid decline in lean body mass. Skeletal muscle, a critical endocrine organ, contributes to the body's metabolic health by secreting cytokines and peptides mediating energy metabolism [63]. The depletion of muscular tissue can lead to various severe outcomes, encompassing weakness, incapacity, and fatality [64, 65]. In contrast, visceral obesity is often accompanied by significant disorders of glucolipid metabolism. It exhibits high insulin resistance, which can have various adverse impacts on the body, leading to a lower renal clearance of uric acid and elevated SUA [66]. The current investigation revealed that hyperuricemia declined following the In LMI/VFMI increase. This correlation remained consistent regardless of the varied attributes of the participants involved in the research. Additional examination of the curve fit indicated a nonlinear connection between the index and hyperuricemia, demonstrating a saturation effect. These findings emphasize the significance of preserving the equilibrium between muscle and visceral fat in human individuals.

Current methods of assessing obesity using BMI and WC may not accurately reflect an individual's obesity status and the distribution of muscle and fat [35]. Research has shown that relying solely on BMI may overlook cardiometabolic risks in individuals with normal BMI but excessive body fat [67]. Our study revealed a strong positive correlation between LMI, VFMI, and hyperuricemia across different levels of BMI and WC. When considering the effects of lean body and visceral fat mass, the correlation between In LMI/VFMI and hyperuricemia remained consistent across different BMI and WC strata, with no statistically significant differences between the subgroups (P for interaction all >0.05). These findings highlight the intricate relationship between muscle mass, adipose tissue, and metabolic health. Neglecting the role of adipose tissue in studying the association between muscle mass and SUA may lead to an incomplete assessment of these variables. In particular, the threshold effect between In LMI/VFMI and hyperuricemia reinforces this conjecture. Evaluating the ratio of LMI to VFMI could provide valuable insights beyond traditional obesity assessment methods.

Certain limitations exist in the present research. Initially, this study adopted a cross-sectional design, thus preventing the establishment of causal relationships among the variables. Cohort studies are needed to determine whether a specific muscle visceral fat ratio range implies better metabolic health. Secondly, it is worth mentioning that the NHANES survey only measured body composition in individuals aged up to 59 years. Therefore, this study's findings may not apply to people older than 59. Another consideration is the potential impact of blood uric acid-lowering medications on the results of this study. Although participants with gout were excluded from the sensitivity analyses, bias from potential confounders is still possible.

## Conclusions

To summarize, the present investigation has revealed a positive association between LMI and VFMI with hyperuricemia. Furthermore, we have observed a non-linear inverse relationship, referred to as the saturation effect, between In LMI/VFMI and hyperuricemia. These findings propose that the LMI/VFMI ratio may offer valuable perspectives beyond solely evaluating separate indicators of lean or visceral fat mass.

### Supplementary Information

**Supplementary Material 1.** 

**Supplementary Material 2.** 

**Supplementary Material 3.** 

**Supplementary Material 4.** 

**Supplementary Material 5.** 

**Supplementary Material 6.** 

**Supplementary Material 7.** 

## Data Availability

The data for this research can be accessed on the following websites: https://www.cdc.gov/nchs/nhanes/about_nhanes.htm.

## Abbreviations

ALT: Alanine aminotransferase
BMI: Body mass index
CVD: Cardiovascular disease
eGFR: Estimated Glomerular filtration rate
LMI: Lean body mass index
MET: Metabolic equivalent
MICE: Multiple imputations by chained equation
NHANES: National Health and Nutrition Examination Survey
SD: Standard deviation
SUA: Serum uric acid
TC: Total cholesterol
VFMI: Visceral fat mass index
WC: Waist circumference
