# Supplementary material for: L-shaped association between lean body mass to visceral fat mass ratio with hyperuricemia: a cross-sectional study
Source: Lipids Health Dis. 2024 Apr 20;23:116. doi: 10.1186/s12944-024-02111-2 (PMC11032594; doi:10.1186/s12944-024-02111-2)
Supplement: Supplementary file 2 — Supplementary Material 2. [file 12944_2024_2111_MOESM2_ESM.docx]

**Supplement Table 1** sensitivity analysis for the relationship between lean body mass index, visceral fat mass index and lean body mass to visceral fat mass ratio with hyperuricemia (***SUA≥6.8mmol/L***)

| **Independent variables** | **Model 1 (n=9646)** | |  | **Model 2 (n=9646)** | |  | **Model 3 (n=9646)** | |
| --- | --- | --- | --- | --- | --- | --- | --- | --- |
|  | OR (95%CI) | *P*-value |  | OR(95%CI) | *P*-value |  | OR (95%CI) | *P*-value |
| **LMI** |  |  |  |  |  |  |  |  |
| Per-SD | 2.40 (2.25, 2.55) | <0.001 |  | 1.76 (1.63, 1.89) | <0.001 |  | 1.76 (1.64, 1.90) | <0.001 |
| Q1 | 1.00 (ref) |  |  | 1.00 (ref) |  |  | 1.00 (ref) |  |
| Q2 | 3.69 (2.79, 4.87) | <0.001 |  | 1.98 (1.48, 2.66) | <0.001 |  | 1.98 (1.48, 2.65) | <0.001 |
| Q3 | 6.53 (5.00, 8.52) | <0.001 |  | 2.72 (2.04, 3.61) | <0.001 |  | 2.72 (2.04, 3.62) | <0.001 |
| Q4 | 14.74 (11.39, 19.08) | <0.001 |  | 5.08 (3.83, 6.75) | <0.001 |  | 5.11 (3.85, 6.78) | <0.001 |
| *P* for trend | <0.001 |  |  | <0.001 |  |  | <0.001 |  |
| **VFMI** |  |  |  |  |  |  |  |  |
| Per-SD | 1.42 (1.35, 1.50) | <0.001 |  | 2.01 (1.86, 2.16) | <0.001 |  | 2.03 (1.88, 2.19) | <0.001 |
| Q1 | 1.00 (ref) |  |  | 1.00 (ref) |  |  | 1.00 (ref) |  |
| Q2 | 2.21 (1.81, 2.70) | <0.001 |  | 2.73 (2.20, 3.38) | <0.001 |  | 2.72 (2.19, 3.37) | <0.001 |
| Q3 | 3.20 (2.65, 3.88) | <0.001 |  | 4.93 (3.96, 6.15) | <0.001 |  | 4.97 (3.98, 6.19) | <0.001 |
| Q4 | 3.49 (2.89, 4.23) | <0.001 |  | 7.92 (6.23, 10.06) | <0.001 |  | 8.03 (6.31, 10.20) | <0.001 |
| *P* for trend | <0.001 |  |  | <0.001 |  |  | <0.001 |  |
| **Ln LMI/VMI** |  |  |  |  |  |  |  |  |
| Per-SD | 0.81 (0.76, 0.86) | <0.001 |  | 0.46 (0.42, 0.50) | <0.001 |  | 0.45 (0.41, 0.50) | <0.001 |
| Q1 | 1.00 (ref) |  |  | 1.00 (ref) |  |  | 1.00 (ref) |  |
| Q2 | 1.21 (1.04, 1.41) | 0.014 |  | 0.66 (0.55, 0.78) | <0.001 |  | 0.65 (0.55, 0.78) | <0.001 |
| Q3 | 1.10 (0.94, 1.29) | 0.218 |  | 0.47 (0.39, 0.56) | <0.001 |  | 0.46 (0.38, 0.56) | <0.001 |
| Q4 | 0.57 (0.48, 0.69) | <0.001 |  | 0.18 (0.14, 0.22) | <0.001 |  | 0.17 (0.14, 0.22) | <0.001 |
| *P* for trend | <0.001 |  |  | <0.001 |  |  | <0.001 |  |

Abbreviations: OR Odds Ratio, SD standard deviation, CI confidence interval, LMI lean body mass index, VFMI visceral fat mass index.

Model 1: no variables adjusted. Model 2: sex, age and race were adjusted. Model 3: sex, age, race, education level, PIR, MET scores, alcohol consumption, and smoking were adjusted.

**Supplement Table 2** sensitivity analysis for the relationship between lean body mass index, visceral fat mass index and lean body mass to visceral fat mass ratio with SUA (***SUA as a continuous variable***).

| **Independent variables** | **Model 1 (n=9646)** | |  | **Model 2 (n=9646)** | |  | **Model 3 (n=9646)** | |
| --- | --- | --- | --- | --- | --- | --- | --- | --- |
|  | β(95%CI) | *P*-value |  | β(95%CI) | *P*-value |  | β(95%CI) | *P*-value |
| **LMI** |  |  |  |  |  |  |  |  |
| Per-SD | 0.63 (0.61, 0.66) | <0.001 |  | 0.38 (0.35, 0.41) | <0.001 |  | 0.38 (0.36, 0.41) | <0.001 |
| Q1 | 1.00 (ref) |  |  | 1.00 (ref) |  |  | 1.00 (ref) |  |
| Q2 | 0.63 (0.57, 0.70) | <0.001 |  | 0.33 (0.27, 0.40) | <0.001 |  | 0.34 (0.27, 0.40) | <0.001 |
| Q3 | 1.08 (1.01, 1.15) | <0.001 |  | 0.58 (0.51, 0.65) | <0.001 |  | 0.59 (0.52, 0.66) | <0.001 |
| Q4 | 1.67 (1.60, 1.74) | <0.001 |  | 0.97 (0.89, 1.04) | <0.001 |  | 0.97 (0.90, 1.05) | <0.001 |
| *P* for trend | <0.001 |  |  | <0.001 |  |  | <0.001 |  |
| **VFMI** |  |  |  |  |  |  |  |  |
| Per-SD | 0.29 (0.27, 0.32) | <0.001 |  | 0.42 (0.39, 0.44) | <0.001 |  | 0.43 (0.40, 0.45) | <0.001 |
| Q1 | 1.00 (ref) |  |  | 1.00 (ref) |  |  | 1.00 (ref) |  |
| Q2 | 0.40 (0.32, 0.47) | <0.001 |  | 0.42 (0.35, 0.48) | <0.001 |  | 0.42 (0.35, 0.48) | <0.001 |
| Q3 | 0.67 (0.59, 0.74) | <0.001 |  | 0.76 (0.69, 0.83) | <0.001 |  | 0.77 (0.70, 0.84) | <0.001 |
| Q4 | 0.80 (0.72, 0.87) | <0.001 |  | 1.10 (1.03, 1.18) | <0.001 |  | 1.12 (1.05, 1.20) | <0.001 |
| *P* for trend | <0.001 |  |  | <0.001 |  |  | <0.001 |  |
| **Ln LMI/VMI** |  |  |  |  |  |  |  |  |
| Per-SD | -0.17 (-0.19, -0.14) | <0.001 |  | -0.39 (-0.42, -0.36) | <0.001 |  | -0.40 (-0.42, -0.37) | <0.001 |
| Q1 | 1.00 (ref) |  |  | 1.00 (ref) |  |  | 1.00 (ref) |  |
| Q2 | 0.08 (0.01, 0.16) | 0.035 |  | -0.31 (-0.37, -0.24) | <0.001 |  | -0.32 (-0.38, -0.25) | <0.001 |
| Q3 | -0.02 (-0.10, 0.05) | 0.564 |  | -0.54 (-0.61, -0.47) | <0.001 |  | -0.55 (-0.62, -0.48) | <0.001 |
| Q4 | -0.35 (-0.43, -0.28) | <0.001 |  | -0.97 (-1.05, -0.90) | <0.001 |  | -0.99 (-1.07, -0.91) | <0.001 |
| *P* for trend | <0.001 |  |  | <0.001 |  |  | <0.001 |  |

Abbreviations: SD standard deviation, CI confidence interval, LMI lean body mass index, VFMI visceral fat mass index.

Model 1: no variables adjusted. Model 2: sex, age and race were adjusted. Model 3: sex, age, race, education level, PIR, MET scores, alcohol consumption, and smoking were adjusted.

**Supplement Table 3** sensitivity analysis for the relationship between lean body mass index, visceral fat mass index and lean body mass to visceral fat mass ratio with hyperuricemia (***Missing data without multiple imputation by chained equation***).

| **Independent variables** | **Model 1 (n=9646)** | |  | **Model 2 (n=9646)** | |  | **Model 3 (n=2767)** | |
| --- | --- | --- | --- | --- | --- | --- | --- | --- |
|  | OR (95%CI) | *P*-value |  | OR(95%CI) | *P*-value |  | OR (95%CI) | *P*-value |
| **LMI** |  |  |  |  |  |  |  |  |
| Per-SD | 1.91 (1.80, 2.02) | <0.001 |  | 1.87 (1.74, 2.00) | <0.001 |  | 1.84 (1.61, 2.11) | <0.001 |
| Q1 | 1.00 (ref) |  |  | 1.00 (ref) |  |  | 1.00 (ref) |  |
| Q2 | 1.90 (1.54, 2.35) | <0.001 |  | 1.81 (1.45, 2.25) | <0.001 |  | 1.37 (0.87, 2.14) | 0.173 |
| Q3 | 3.07 (2.51, 3.75) | <0.001 |  | 2.78 (2.24, 3.45) | <0.001 |  | 2.36 (1.51, 3.67) | <0.001 |
| Q4 | 5.91 (4.88, 7.15) | <0.001 |  | 5.34 (4.29, 6.64) | <0.001 |  | 4.45 (2.84, 6.96) | <0.001 |
| *P* for trend | <0.001 |  |  | <0.001 |  |  | <0.001 |  |
| **VFMI** |  |  |  |  |  |  |  |  |
| Per-SD | 1.59 (1.51, 1.67) | <0.001 |  | 1.99 (1.86, 2.13) | <0.001 |  | 1.99 (1.74, 2.27) | <0.001 |
| Q1 | 1.00 (ref) |  |  | 1.00 (ref) |  |  | 1.00 (ref) |  |
| Q2 | 2.18 (1.78, 2.68) | <0.001 |  | 2.54 (2.06, 3.13) | <0.001 |  | 2.33 (1.62, 3.36) | <0.001 |
| Q3 | 3.32 (2.73, 4.03) | <0.001 |  | 4.56 (3.70, 5.64) | <0.001 |  | 4.22 (2.91, 6.13) | <0.001 |
| Q4 | 4.61 (3.81, 5.58) | <0.001 |  | 8.23 (6.59, 10.28) | <0.001 |  | 6.87 (4.59, 10.30) | <0.001 |
| *P* for trend | <0.001 |  |  | <0.001 |  |  | <0.001 |  |
| **Ln LMI/VMI** |  |  |  |  |  |  |  |  |
| Per-SD | 0.68 (0.64, 0.72) | <0.001 |  | 0.46 (0.42, 0.50) | <0.001 |  | 0.48 (0.40, 0.56) | <0.001 |
| Q1 | 1.00 (ref) |  |  | 1.00 (ref) |  |  | 1.00 (ref) |  |
| Q2 | 0.87 (0.75, 1.01) | 0.064 |  | 0.61 (0.52, 0.71) | <0.001 |  | 0.70 (0.52, 0.96) | 0.025 |
| Q3 | 0.70 (0.61, 0.82) | <0.001 |  | 0.41 (0.34, 0.48) | <0.001 |  | 0.41 (0.29, 0.58) | <0.001 |
| Q4 | 0.35 (0.30, 0.42) | <0.001 |  | 0.16 (0.13, 0.21) | <0.001 |  | 0.20 (0.13, 0.31) | <0.001 |
| *P* for trend | <0.001 |  |  | <0.001 |  |  | <0.001 |  |

Abbreviations: OR Odds Ratio, SD standard deviation, CI confidence interval, LMI lean body mass index, VFMI visceral fat mass index.

Model 1: no variables adjusted. Model 2: sex, age and race were adjusted. Model 3: sex, age, race, education level, PIR, MET scores, alcohol consumption, and smoking were adjusted.

**Supplement Table 4** sensitivity analysis for the relationship between lean body mass index, visceral fat mass index and lean body mass to visceral fat mass ratio with hyperuricemia (***NHANES 2011-2016***)

| **Independent variables** | **Model 1 (n=7679)** | |  | **Model 2 (n=7679)** | |  | **Model 3 (n=7679)** | |
| --- | --- | --- | --- | --- | --- | --- | --- | --- |
|  | OR (95%CI) | *P*-value |  | OR(95%CI) | *P*-value |  | OR (95%CI) | *P*-value |
| **LMI** |  |  |  |  |  |  |  |  |
| Per-SD | 1.91 (1.79, 2.04) | <0.001 |  | 1.87 (1.73, 2.02) | <0.001 |  | 1.87 (1.73, 2.03) | <0.001 |
| Q1 | 1.00 (ref) |  |  | 1.00 (ref) |  |  | 1.00 (ref) |  |
| Q2 | 1.96 (1.54, 2.49) | <0.001 |  | 1.86 (1.45, 2.38) | <0.001 |  | 1.86 (1.45, 2.38) | <0.001 |
| Q3 | 3.10 (2.47, 3.89) | <0.001 |  | 2.80 (2.19, 3.58) | <0.001 |  | 2.80 (2.20, 3.58) | <0.001 |
| Q4 | 5.94 (4.78, 7.38) | <0.001 |  | 5.34 (4.17, 6.85) | <0.001 |  | 5.36 (4.18, 6.88) | <0.001 |
| *P* for trend | <0.001 |  |  | <0.001 |  |  | <0.001 |  |
| **VFMI** |  |  |  |  |  |  |  |  |
| Per-SD | 1.59 (1.50, 1.68) | <0.001 |  | 2.04 (1.89, 2.20) | <0.001 |  | 2.06 (1.91, 2.22) | <0.001 |
| Q1 | 1.00 (ref) |  |  | 1.00 (ref) |  |  | 1.00 (ref) |  |
| Q2 | 2.40 (1.90, 3.03) | <0.001 |  | 2.82 (2.22, 3.59) | <0.001 |  | 2.82 (2.22, 3.58) | <0.001 |
| Q3 | 3.56 (2.84, 4.46) | <0.001 |  | 5.07 (3.98, 6.47) | <0.001 |  | 5.09 (3.99, 6.49) | <0.001 |
| Q4 | 4.87 (3.91, 6.06) | <0.001 |  | 9.25 (7.16, 11.93) | <0.001 |  | 9.35 (7.24, 12.08) | <0.001 |
| *P* for trend | <0.001 |  |  | <0.001 |  |  | <0.001 |  |
| **Ln LMI/VMI** |  |  |  |  |  |  |  |  |
| Per-SD | 0.67 (0.63, 0.72) | <0.001 |  | 0.44 (0.40, 0.49) | <0.001 |  | 0.44 (0.40, 0.48) | <0.001 |
| Q1 | 1.00 (ref) |  |  | 1.00 (ref) |  |  | 1.00 (ref) |  |
| Q2 | 0.83 (0.71, 0.98) | 0.031 |  | 0.56 (0.47, 0.67) | <0.001 |  | 0.56 (0.47, 0.67) | <0.001 |
| Q3 | 0.72 (0.60, 0.85) | <0.001 |  | 0.40 (0.33, 0.48) | <0.001 |  | 0.39 (0.32, 0.48) | <0.001 |
| Q4 | 0.34 (0.28, 0.42) | <0.001 |  | 0.15 (0.12, 0.19) | <0.001 |  | 0.15 (0.11, 0.19) | <0.001 |
| *P* for trend | <0.001 |  |  | <0.001 |  |  | <0.001 |  |

Abbreviations: OR Odds Ratio, SD standard deviation, CI confidence interval, LMI lean body mass index, VFMI visceral fat mass index.

Model 1: no variables adjusted. Model 2: sex, age and race were adjusted. Model 3: sex, age, race, education level, PIR, MET scores, alcohol consumption, and smoking were adjusted.

**Supplement Table 5** sensitivity analysis for the relationship between lean body mass index, visceral fat mass index and lean body mass to visceral fat mass ratio with hyperuricemia (***excluding participants with gout***)

| **Independent variables** | **Model 1 (n=9430)** | |  | **Model 2 (n=9430)** | |  | **Model 3 (n=9430)** | |
| --- | --- | --- | --- | --- | --- | --- | --- | --- |
|  | OR (95%CI) | *P*-value |  | OR(95%CI) | *P*-value |  | OR (95%CI) | *P*-value |
| **LMI** |  |  |  |  |  |  |  |  |
| Per-SD | 1.89 (1.78, 2.01) | <0.001 |  | 1.87 (1.74, 2.01) | <0.001 |  | 1.88 (1.75, 2.02) | <0.001 |
| Q1 | 1.00 (ref) |  |  | 1.00 (ref) |  |  | 1.00 (ref) |  |
| Q2 | 1.92 (1.55, 2.39) | <0.001 |  | 1.85 (1.48, 2.32) | <0.001 |  | 1.86 (1.49, 2.32) | <0.001 |
| Q3 | 3.03 (2.47, 3.71) | <0.001 |  | 2.81 (2.26, 3.50) | <0.001 |  | 2.82 (2.27, 3.52) | <0.001 |
| Q4 | 5.77 (4.75, 7.02) | <0.001 |  | 5.36 (4.29, 6.71) | <0.001 |  | 5.39 (4.31, 6.75) | <0.001 |
| *P* for trend | <0.001 |  |  | <0.001 |  |  | <0.001 |  |
| **VFMI** |  |  |  |  |  |  |  |  |
| Per-SD | 1.58 (1.49, 1.66) | <0.001 |  | 2.00 (1.87, 2.14) | <0.001 |  | 2.02 (1.89, 2.17) | <0.001 |
| Q1 | 1.00 (ref) |  |  | 1.00 (ref) |  |  | 1.00 (ref) |  |
| Q2 | 2.16 (1.76, 2.66) | <0.001 |  | 2.55 (2.06, 3.16) | <0.001 |  | 2.55 (2.06, 3.16) | <0.001 |
| Q3 | 3.31 (2.71, 4.04) | <0.001 |  | 4.66 (3.75, 5.78) | <0.001 |  | 4.71 (3.80, 5.85) | <0.001 |
| Q4 | 4.51 (3.71, 5.48) | <0.001 |  | 8.32 (6.63, 10.45) | <0.001 |  | 8.50 (6.76, 10.68) | <0.001 |
| *P* for trend | <0.001 |  |  | <0.001 |  |  | <0.001 |  |
| **Ln LMI/VMI** |  |  |  |  |  |  |  |  |
| Per-SD | 0.68 (0.64, 0.72) | <0.001 |  | 0.46 (0.42, 0.50) | <0.001 |  | 0.45 (0.41, 0.49) | <0.001 |
| Q1 | 1.00 (ref) |  |  | 1.00 (ref) |  |  | 1.00 (ref) |  |
| Q2 | 0.88 (0.76, 1.03) | 0.105 |  | 0.61 (0.52, 0.72) | <0.001 |  | 0.61 (0.52, 0.72) | <0.001 |
| Q3 | 0.72 (0.62, 0.84) | <0.001 |  | 0.41 (0.34, 0.49) | <0.001 |  | 0.40 (0.34, 0.48) | <0.001 |
| Q4 | 0.36 (0.30, 0.43) | <0.001 |  | 0.16 (0.13, 0.21) | <0.001 |  | 0.16 (0.13, 0.20) | <0.001 |
| *P* for trend | <0.001 |  |  | <0.001 |  |  | <0.001 |  |

Abbreviations: OR Odds Ratio, SD standard deviation, CI confidence interval, LMI lean body mass index, VFMI visceral fat mass index.

Model 1: no variables adjusted. Model 2: sex, age and race were adjusted. Model 3: sex, age, race, education level, PIR, MET scores, alcohol consumption, and smoking were adjusted.
