# Supplementary figures and images for: L-shaped association between lean body mass to visceral fat mass ratio with hyperuricemia: a cross-sectional study
Source: Lipids Health Dis. 2024 Apr 20;23:116. doi: 10.1186/s12944-024-02111-2 (PMC11032594; doi:10.1186/s12944-024-02111-2)

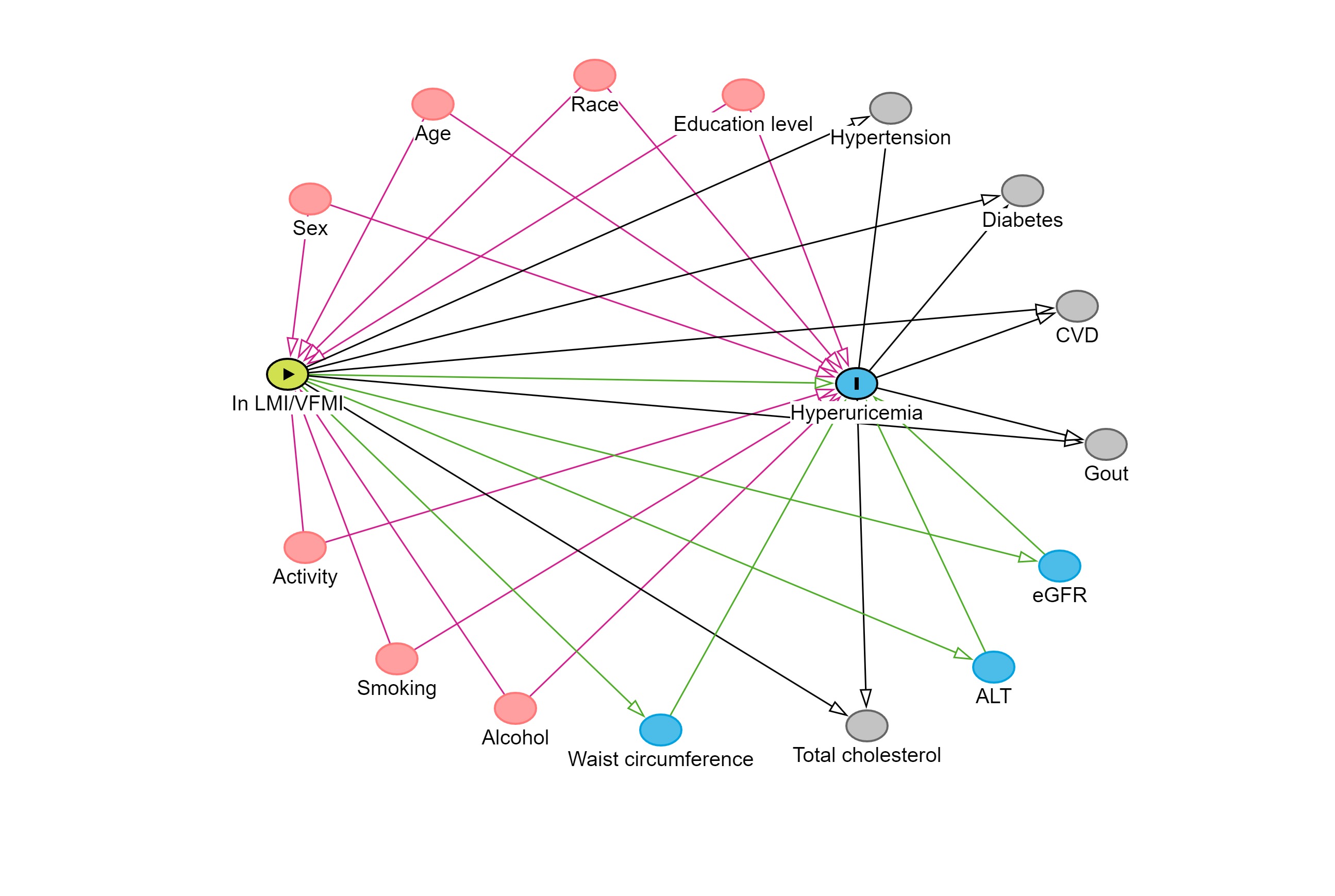

Supplement: Supplementary file 3 — Supplementary Material 3. [file 12944_2024_2111_MOESM3_ESM.jpeg]

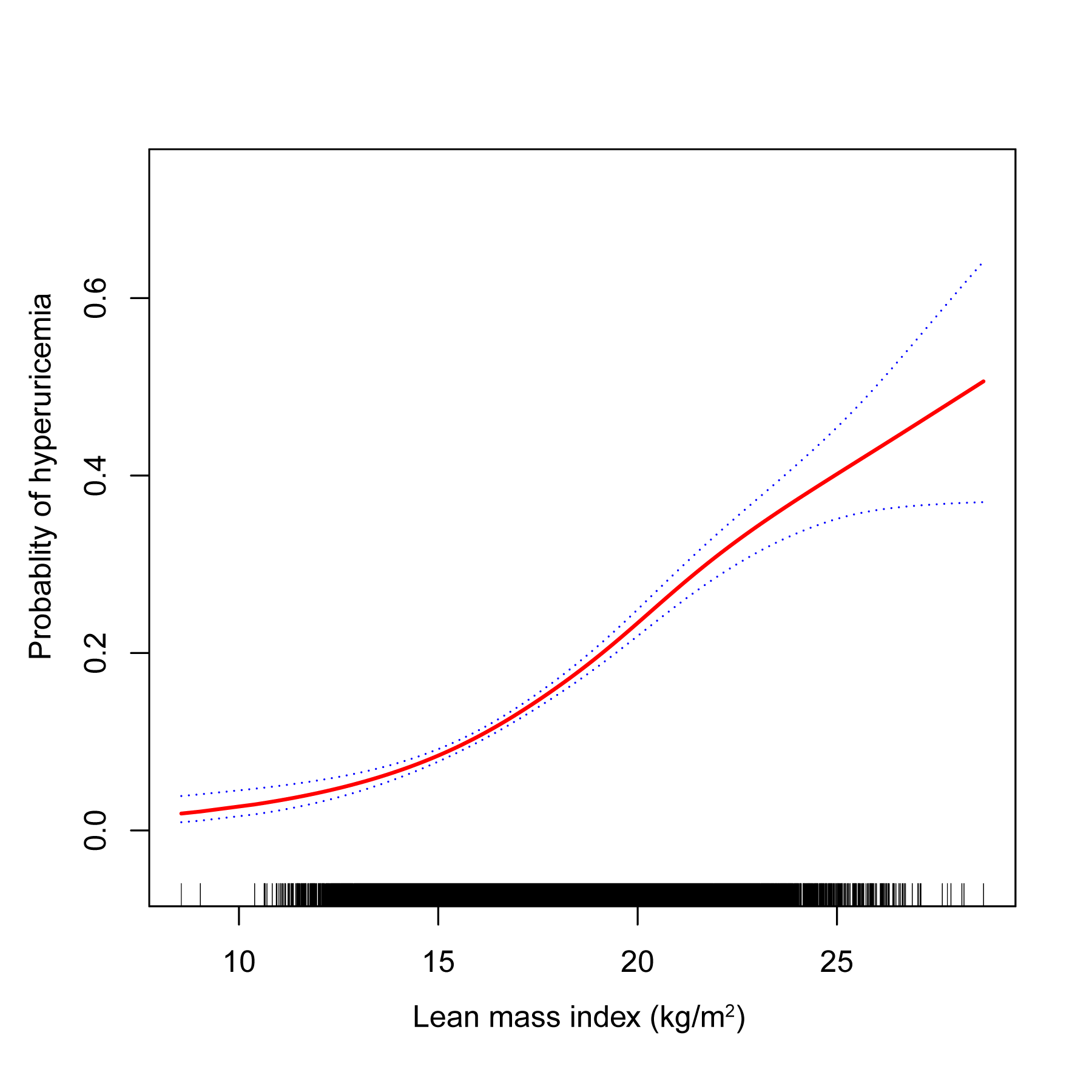

Supplement: Supplementary file 4 — Supplementary Material 4. [file 12944_2024_2111_MOESM4_ESM.tif]

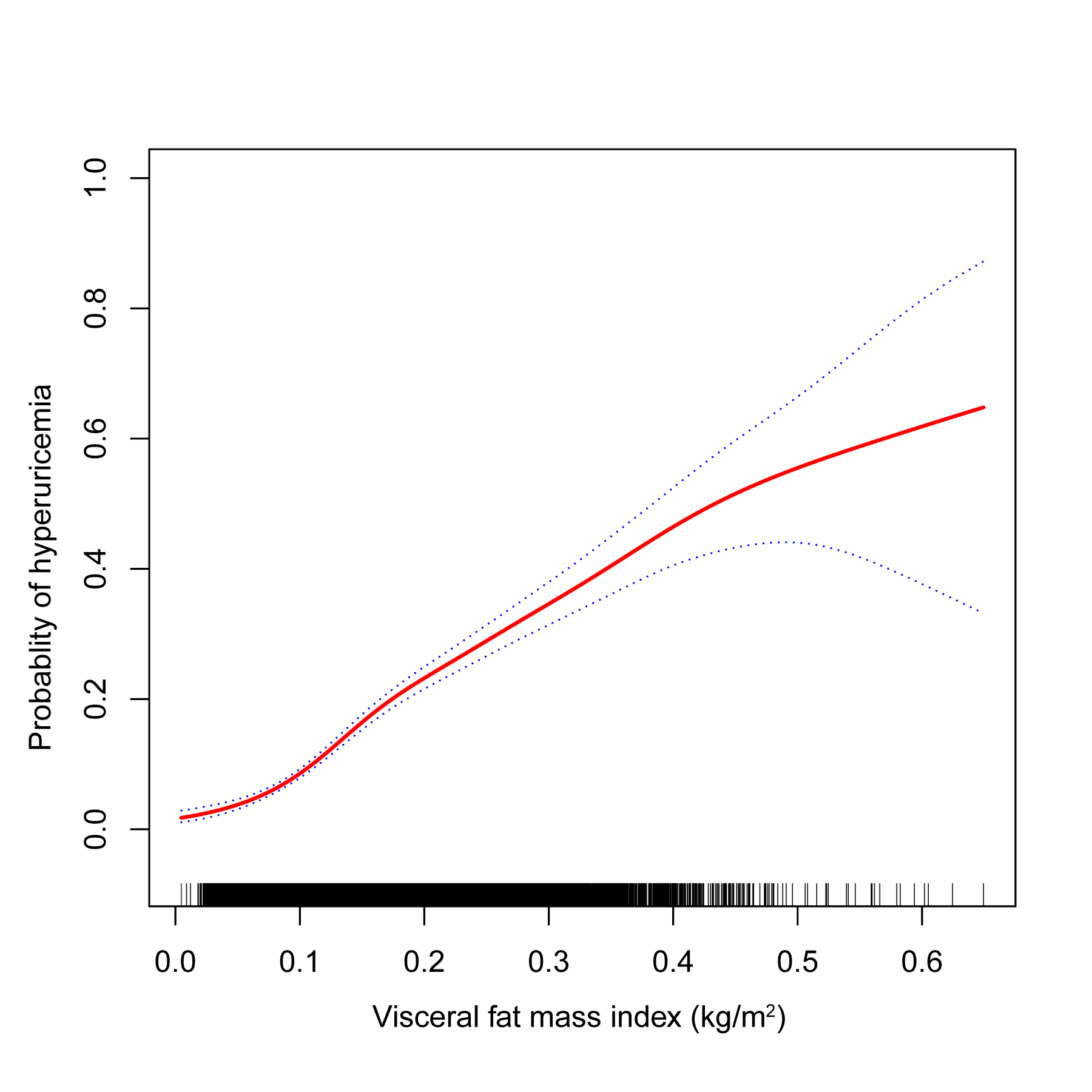

Supplement: Supplementary file 5 — Supplementary Material 5. [file 12944_2024_2111_MOESM5_ESM.tif]

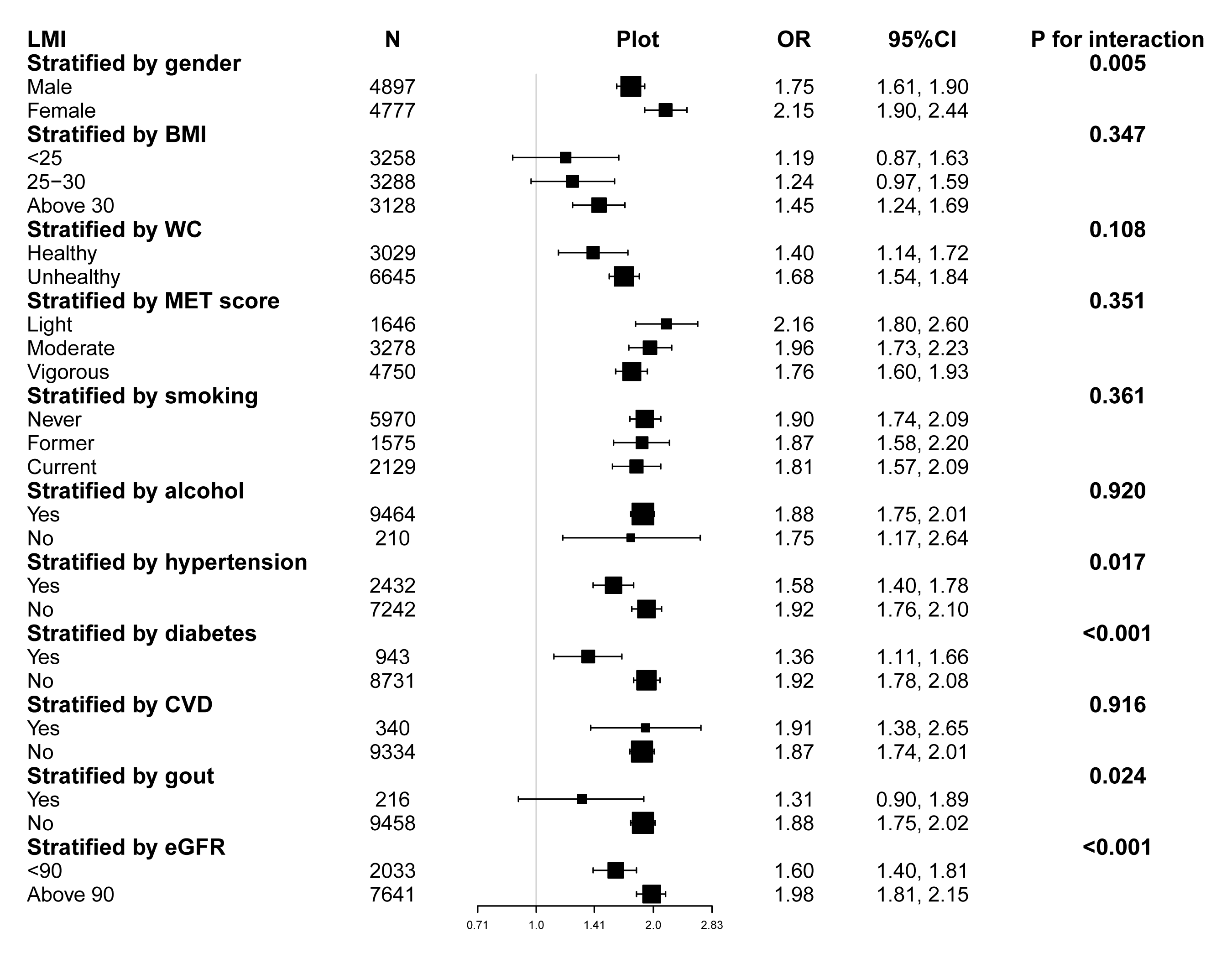

Supplement: Supplementary file 6 — Supplementary Material 6. [file 12944_2024_2111_MOESM6_ESM.tif]

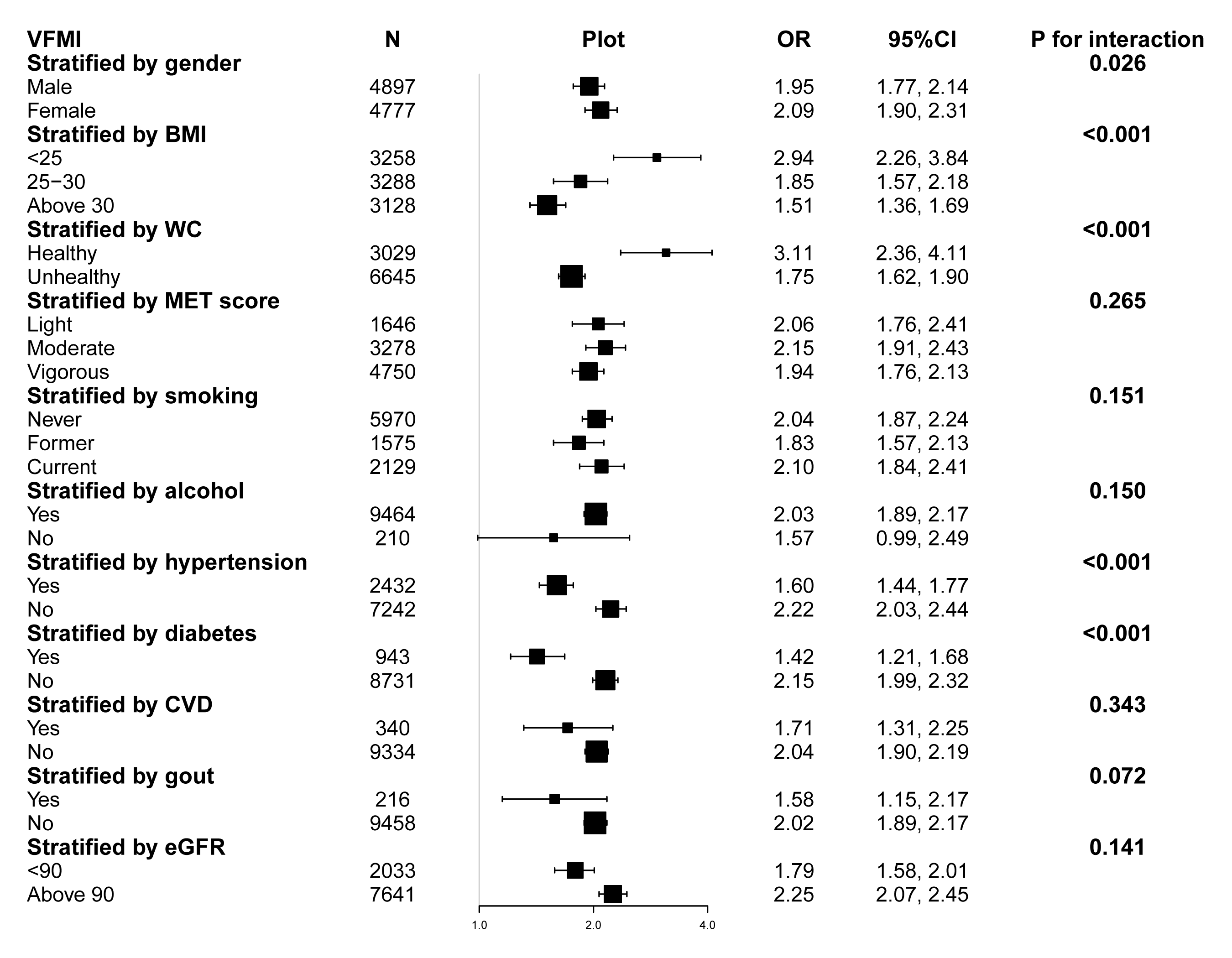

Supplement: Supplementary file 7 — Supplementary Material 7. [file 12944_2024_2111_MOESM7_ESM.tif]
